# Benefits of specialist severe asthma management: demographic and geographic disparities

**DOI:** 10.1183/13993003.00660-2022

**Published:** 2022-12-15

**Authors:** Charlene Redmond, Liam G. Heaney, Rekha Chaudhuri, David J. Jackson, Andrew Menzies-Gow, Paul Pfeffer, John Busby

**Affiliations:** 1 School of Medicine, Dentistry and Biomedical Sciences, Queen's University, Belfast, UK; 2 Belfast Health and Social Care NHS Trust, Belfast, UK; 3 Gartnavel General Hospital, Glasgow, UK; 4 Guy's Severe Asthma Centre, Guy's and St Thomas’ Hospitals, London, UK; 5 School of Immunology and Microbial Sciences, King's College London, London, UK; 6 Royal Brompton and Harefield Hospitals, London, UK; 7 Barts Health NHS Trust, London, UK

## Abstract

**Background:**

The benefits of specialist assessment and management have yet to be evaluated within the biologic era of UK severe asthma treatment, and potential disparities have not been considered.

**Methods:**

In an uncontrolled before-and-after study, we compared asthma symptoms (Asthma Control Questionnaire-6 (ACQ-6)), exacerbations, unscheduled secondary care use, lung function (forced expiratory volume in 1 s (FEV_1_)) and oral corticosteroid (OCS) dose after 1 year. We compared outcomes by sex, age (18–34, 35–49, 50–64 and ≥65 years), ethnicity (Caucasian *versus* non-Caucasian) and hospital site after adjusting for demographics and variation in biologic therapy use.

**Results:**

1140 patients were followed-up for 1370 person-years from 12 specialist centres. At annual review, ACQ-6 score was reduced by a median (interquartile range (IQR)) of 0.7 (0.0–1.5), exacerbations by 75% (33–100%) and unscheduled secondary care by 100% (67–100%). FEV_1_ increased by a median (IQR) of 20 (−200–340) mL, while OCS dose decreased for 67% of patients. Clinically meaningful improvements occurred across almost all patients, including those not receiving biologic therapy. There was little evidence of differences across demographic groups, although those aged ≥65 years demonstrated larger reductions in exacerbations (69% *versus* 52%; p<0.001) and unscheduled care use (77% *versus* 50%; p<0.001) compared with patients aged 18–34 years. There were >2-fold differences between the best and worst performing centres across all study outcomes.

**Conclusions:**

Specialist assessment and management is associated with substantially improved patient outcomes, which are broadly consistent across demographic groups and are not restricted to those receiving biologic therapy. Significant variation exists between hospitals, which requires further investigation.

## Abstract 2

**Specialist assessment and management of patients with severe asthma leads to substantially improved patient outcomes, which are broadly consistent across demographic groups although vary substantially across hospitals** https://bit.ly/3ORDfei

## Introduction

Asthma is a heterogeneous respiratory disease estimated to affect over 330 million people worldwide [1], with ∼5% of these having severe disease [2]. Severe asthma is defined as asthma that remains uncontrolled despite patient adherence to optimised therapies (high-dose inhaled corticosteroids, long-acting β-agonists and treatment of contributory factors) or asthma which worsens when high-dose treatment is reduced/stepped down [3]. The British Thoracic Society (BTS) and European Respiratory Society (ERS)/American Thoracic Society (ATS) have highlighted the need for patients with difficult or severe asthma to be systematically evaluated by specialists [4, 5]. Within the UK, care for patients with severe asthma is currently provided by specialists based within regional centres.

There has been much interest investigating the benefits of specialist assessment and management since it was recommended for difficult to control asthma over 30 years ago [6]. Studies in the USA, France, Australia and the Netherlands [7–13] have demonstrated that specialist assessment can lead to improved asthma control, reduced exacerbations and a lowering of maintenance oral corticosteroid (mOCS) dose. These findings have been replicated within several UK-based studies and, most recently, Gibeon *et al.* [14] demonstrated that dedicated severe asthma centres led to a significant improvement in asthma symptoms (median Asthma Control Questionnaire (ACQ) score from 3.4 to 2.8), a reduction in the proportion of patients admitted to hospital (48% *versus* 38%) and a lowering of mOCS dose (15 *versus* 10 mg). Crucially, only 12% of this study cohort were receiving biologic therapy as this analysis used data collected from patients registered during 2009 and 2010. Recent evidence from the UK and elsewhere has highlighted potential disparities in asthma morbidity based on patient gender, ethnicity and socioeconomic status [15–18]. However, the moderating impact of demographic and geographic factors on outcomes among those treated in specialist asthma centres has yet to be assessed. Consequently, an updated analysis is required to estimate the benefits of specialist assessment for people with severe asthma in the “biologic era” and to assess if these benefits are observed equally across patient groups.

The primary aim of this study is to estimate the benefits of specialist assessment of patients with severe asthma. As a secondary objective we investigate if these benefits are observed equally across patient groups and seek to identify evidence of unmet need.

## Methods

### Study population and design

The UK Severe Asthma Registry (UKSAR) collects standardised data on patients with severe asthma that have been referred to specialist services in England, Scotland and Northern Ireland. Variables contained within the dataset include demographic characteristics, patient medical history, current treatment regimes, lung function and inflammatory biomarkers. Further details about the registry can be found elsewhere [19, 20]. The UKSAR has database ethical approval from the Office of Research Ethics Northern Ireland (15/NI/0196) and all patients provide written informed consent. All patients in this analysis were first seen during 2016–2020 and assessed as meeting ERS/ATS severe asthma guidelines [19]. Patients were included if they were aged ≥18 years at first assessment, had at least one annual review (within 9–24 months of their baseline assessment) and were not receiving biologic therapy at the time of their baseline assessment. This was an uncontrolled before-and-after study.

### Exposures, outcomes and covariates

Comparisons were made between baseline and first annual review visit. The outcomes of interest were ACQ-6 improvement (score), exacerbation reduction (%), reduction in unscheduled care utilisation (%), forced expiratory volume in 1 s (FEV_1_) improvement (mL) and OCS discontinuation (%). Exacerbation reduction compared the number of times the patient required rescue corticosteroids in the 12 months prior to baseline against the 12 months prior to the annual review visit. Unscheduled care reduction was also based on the 12 months prior to the relevant study visit, and was a composite measure of emergency department (ED) attendances and hospital admissions. The OCS discontinuation outcome was restricted to patients on mOCS at baseline and was defined as not receiving any mOCS at the time of their annual review. We created a composite measure to quantify the distribution of improvements across patients. Patients were categorised according to the number of domains in which they had a material improvement in over the study period: asthma symptoms (ACQ-6 improvement ≥0.5 or well controlled (ACQ-6 ≤0.75) at annual review), exacerbations (reduction ≥50% or no exacerbations in the 12 months prior to annual review), unscheduled care (reduction ≥50% or no unscheduled care in the 12 months prior to annual review), FEV_1_ (≥100 mL improvement) and OCS (dose reduction ≥50% or not a mOCS user at annual review).

Our secondary objective compared improvement across groups based on age (18–34, 35–49, 50–64 and ≥65 years), sex (female *versus* male) and ethnicity. The ethnicity of patients in the UKSAR is recorded according to Global Lung Initiative criteria, although to increase statistical power in the current study we made comparisons between Caucasian (White) and non-Caucasian (Southeast Asian, Northeast Asian, African, Mixed and Other) patients [16]. We compared improvements by biologic prescription and assessed the reason for patients not progressing to biologic therapy by applying current National Institute for Health and Care Excellence (NICE) access criteria which are used across the UK. We also compared outcomes among hospitals to explore geographic variation. To ensure an acceptable level of precision around the hospital-specific estimates, sites were only included in this analysis if they had at least 30 eligible patients in the analysis. Consequently, each analysis had a different number of hospitals included.

### Statistical analyses

Our primary analyses compared the change in study outcomes between baseline and first annual review visit. Descriptive statistics were calculated using mean with standard deviation, median (interquartile range (IQR)) and count (percentage) as appropriate. Differences in characteristics between patients that did, or did not, receive biologic treatment were tested for statistical significance using the Chi-squared test, t-test and Wilcoxon–Mann–Whitney test as appropriate.

For our secondary objective we used hierarchical linear (ACQ-6 score and FEV_1_), Poisson (exacerbations and unscheduled care) and logistic (OCS discontinuation) regression analysis to compare improvements by age, sex, ethnicity and hospital site. Adjusted models included time period (follow-up *versus* annual review), year of baseline visit, hospital, age, sex, ethnicity, biologic prescription prior to annual review and baseline value of the metric (*e.g.* baseline ACQ-6 score, baseline OCS dose). Each patient was included as a random intercept in the model, which allowed their responses to vary and appropriately accounted for the non-independence of observations. Crucially, our models included an interaction term between time period and each candidate demographic/geographic variable, which captured variation in improvement across the groups of interest. Model coefficients were converted to adjusted predictions that represent the improvement in each group of interest, assuming all other variables in the model were fixed [21]. All analyses were conducted under a complete-case framework using Stata version 16 (StataCorp, College Station, TX, USA).

### Sensitivity analyses

We re-ran our regression models omitting the biologic therapy variable to investigate the importance of differing biologic prescription pattern across demographic groups in driving potential disparities. Recent evidence has shown that a substantial proportion of patients experience adrenal insufficiency when tapering their mOCS dose [22], therefore we repeated our analysis classifying OCS discontinuance as complete withdrawal of mOCS or remaining on ≤5 mg at annual review.

## Results

### Patient characteristics

1140 patients were followed-up for 1370 person-years from 12 specialist centres. The median (IQR) time between their baseline and annual review visit was 406 (363–497) days. Data completeness was generally good, although a lower percentage of patients had FEV_1_ (83.8%) and ACQ-6 score (81.5%) available at follow-up (supplementary table E1). The majority of our patient cohort were female (61.1%), with an average age at first assessment of 50.6 years. Most patients were Caucasian (80.4%) and had never smoked (67.7%). At baseline, patients exhibited substantial morbidity, including high symptom burden (median (IQR) ACQ-6 score 3.0 (2.0–4.0)), impaired lung function (mean FEV_1_ 66.6% predicted) and frequent exacerbations in the prior year (median (IQR) 5 (3–8)). A substantial proportion of patients attended the ED (38.9%) and/or were hospitalised (38.8%) for their asthma in the year prior to assessment. Biomarkers of type 2 inflammation, including blood eosinophils (0.40×10^9^ L^−1^), exhaled nitric oxide fraction (41 ppb) and IgE (154 IU·mL^−1^), were frequently elevated at the time of registration. Over half of patients (56.3%) were receiving mOCS at baseline (median dose 10 mg), while the majority (81.3%) progressed to biologics by the time of their annual review visit (table 1). Four different biologic therapies were prescribed to patients in this cohort, with the majority receiving mepolizumab (65.7%), benralizumab (19.7%) or omalizumab (14.4%) (table 1). Over 40% of those who did not receive biologic therapy failed to meet the UK access criteria set by NICE. Of the remainder, 47% required optimisation of current treatment and 33% had issues with medication adherence.

**TABLE 1:** Baseline patient characteristics, presented for both the entire cohort and stratified by biologic treatment

	**Entire cohort**	**Received biologic**		**p-value**
		**No**	**Yes**	
**Patients**	1140	213	927	
**Age at first assessment (years)**	50.6±14.6	48.2±14.7	51.1±14.5	0.010
**Age of onset (years)**	25.4±19.3	25.2±19.2	25.5±19.3	0.847
**Gender**				0.163
Female	696 (61.1)	139 (65.3)	557 (60.1)	
Male	444 (38.9)	74 (34.7)	370 (39.9)	
**Ethnicity**				0.002
Caucasian	913 (80.4)	155 (72.8)	758 (82.1)	
Non-Caucasian	223 (19.6)	58 (27.2)	165 (17.9)	
**BMI (kg·m^−2^)**	30.7±7.1	30.9±7.7	30.7±6.9	0.725
**Smoking status**				0.004
Never-smoker	756 (67.7)	132 (62.6)	624 (69.0)	
Ex-smoker	321 (28.8)	64 (30.3)	257 (28.4)	
Current smoker	39 (3.5)	15 (7.1)	24 (2.7)	
**Atopic disease**	628 (55.7)	123 (58.3)	505 (55.1)	0.404
**FEV_1_ (L)**	2.00±0.78	2.00±0.73	2.00±0.79	0.944
**FEV_1_ (% pred)**	66.6±21.0	68.3±20.9	66.2±21.0	0.190
**FVC (L)**	3.15±1.02	3.02±0.99	3.18±1.02	0.034
**FVC (% pred)**	84.3±18.9	83.5±18.8	84.5±18.9	0.497
**FEV_1_/FVC**	63.7±18.5	66.5±12.4	63.1±19.5	0.017
**ACQ-6 score**	3.0 (2.0–4.0)	3.0 (2.0–4.0)	3.0 (2.0–3.8)	0.702
**Uncontrolled asthma (ACQ-6 >1.5)**	811 (82.2)	161 (82.6)	650 (82.1)	0.872
**EuroQoL utility**	0.73 (0.48–0.88)	0.73 (0.56–0.86)	0.73 (0.45–0.88)	0.674
**Exacerbations (prior year) (n)**	5 (3–8)	4 (2–6)	5 (3–8)	<0.001
**Any ED attendance (prior year)**	435 (38.9)	98 (47.1)	337 (37.0)	0.007
**Any hospital admission (prior year)**	437 (38.8)	79 (37.4)	358 (39.1)	0.659
**Invasive ventilation (ever)**	117 (10.5)	18 (8.7)	99 (10.9)	0.345
**Eczema**	27 (2.4)	3 (1.4)	24 (2.6)	0.307
**Nasal polyps**	211 (18.5)	27 (12.7)	184 (19.8)	0.015
**Gastro-oesophageal reflux**	207 (18.2)	43 (20.2)	164 (17.7)	0.394
**Depression or anxiety**	109 (9.6)	23 (10.8)	86 (9.3)	0.496
**Blood eosinophil count (×10^9^ L^−1^)**	0.40 (0.20–0.60)	0.30 (0.18–0.50)	0.40 (0.20–0.63)	<0.001
**Highest blood eosinophil count (×10^9^ L^−1^)**	0.68 (0.40–1.05)	0.55 (0.30–0.90)	0.70 (0.46–1.10)	<0.001
***F*_ENO_ (ppb)**	41 (22–73)	36 (19–62)	42 (24–75)	0.007
**IgE (IU·mL^−1^)**	154 (53–438)	147 (51–501)	155 (53–420)	0.866
**mOCS**	639 (56.3)	81 (38.2)	558 (60.5)	<0.001
**mOCS dose (prednisolone-equivalent mg)**	10 (8–15)	10 (5–15)	10 (8–15)	0.049
**ICS dose (BDP-equivalent µg)**	2000 (1600–2000)	2000 (1600–2000)	2000 (1600–2000)	0.005
**LAMA**	646 (57.4)	104 (49.3)	542 (59.2)	0.008
**Theophylline**	279 (24.7)	38 (18.0)	241 (26.2)	0.013
**LTRA**	568 (51.2)	120 (58.5)	448 (49.5)	0.019
**Maintenance macrolides**	82 (7.4)	10 (4.9)	72 (7.9)	0.128
**Nebuliser**	232 (20.7)	38 (18.3)	194 (21.3)	0.335
**Biologic therapy**	927 (81.3)	0 (0.0)	927 (100.0)	
**Biologic therapy medication**				
Mepolizumab	591 (65.7)	0 (0.0)	591 (65.7)	
Benralizumab	177 (19.7)	0 (0.0)	177 (19.7)	
Omalizumab	130 (14.4)	0 (0.0)	130 (14.4)	
Dupilumab	2 (0.2)	0 (0.0)	2 (0.2)	

Data are presented as n, mean±sd, median (interquartile range) or n (%), unless otherwise stated. BMI: body mass index; FEV_1_: forced expiratory volume in 1 s; FVC: forced vital capacity; ACQ-6: Asthma Control Questionnaire-6; ED: emergency department; *F*_ENO_: exhaled nitric oxide fraction; mOCS: maintenance oral corticosteroids; ICS: inhaled corticosteroids; BDP: beclometasone dipropionate; LAMA: long-acting muscarinic antagonists; LTRA: leukotriene receptor antagonists.

### Benefits of specialist severe asthma assessment and management

Significant improvements were achieved across all study outcomes. In particular, the median (IQR) ACQ-6 score was reduced by 0.7 (0.0–1.5), exacerbations reduced by 75% (33–100%) and unscheduled care reduced by 100% (67–100%). mOCS dose was reduced in the majority of patients (67%) and for some patients (20.2%) was discontinued altogether; however, limited changes were noted for FEV_1_ (median (IQR) increase 20 (−200–340) mL) (table 2). A median (IQR) reduction of 80% (28–98%) in blood eosinophils was achieved among patients receiving a biologic compared with no change among those who were not (median (IQR) reduction 0% (−68–50%)). Almost all (97.3%) patients had a material improvement in at least one study outcome and the median (IQR) improvement was n=3 (2–4) outcomes. A substantial minority of patients (15.5%) experienced an improvement across all five outcomes (table 2 and supplementary figure E1).

**TABLE 2:** Patient characteristics at follow-up review, presented for both the entire cohort and stratified by biologic treatment

	**Entire cohort**	**Received biologic**		**p-value**
		**No**	**Yes**	
**Patients**	1140	213	927	
**Follow-up time (days)**	406 (363–497)	413 (357–511)	405 (363–494)	0.586
**BMI (kg·m^−2^)**	31.1±7.2	31.4±8.3	31.0±6.9	0.465
**Exacerbations (prior year) (n)**	1 (0–3)	2 (0–4)	1 (0–3)	0.129
0	371 (33.7)	63 (31.2)	308 (34.3)	
1	205 (18.6)	35 (17.3)	170 (18.9)	
2	152 (13.8)	21 (10.4)	131 (14.6)	
3	109 (9.9)	30 (14.9)	79 (8.8)	
4+	264 (24.0)	53 (26.2)	211 (23.5)	
**Any ED attendance (prior year)**	170 (15.6)	43 (21.4)	127 (14.3)	0.012
**Any hospital admission (prior year)**	187 (17.2)	44 (21.8)	143 (16.1)	0.053
**Blood eosinophil count (×10^9^ L^−1^)**	0.10 (0.01–0.23)	0.30 (0.15–0.50)	0.08 (0.00–0.19)	<0.001
***F*_ENO_ (ppb)**	36 (20–67)	33 (21–55)	36 (20–70)	0.362
**FEV_1_ (L)**	2.10±0.79	2.06±0.75	2.11±0.80	0.458
**FEV_1_ (% pred)**	70.3±20.9	70.6±19.5	70.3±21.2	0.871
**FVC (L)**	3.17±1.03	3.06±1.03	3.19±1.03	0.150
**FVC (% pred)**	84.9±19.3	84.0±18.8	85.2±19.4	0.497
**FEV_1_/FVC**	66.4±13.3	67.9±12.1	66.1±13.6	0.115
**ACQ-6 score**	2.0 (0.8–3.3)	2.8 (1.5–3.8)	1.8 (0.8–3.2)	<0.001
**Uncontrolled asthma (ACQ-6 >1.5)**	556 (59.8)	112 (74.7)	444 (57.0)	<0.001
**EuroQoL utility**	0.76 (0.49–0.92)	0.73 (0.43–0.88)	0.78 (0.50–0.94)	0.175
**mOCS**	587 (51.6)	84 (39.6)	503 (54.3)	<0.001
**mOCS dose (prednisolone-equivalent mg)**	8 (5–13)	10 (5–10)	8 (5–13)	0.270
**Difference from baseline**				
ACQ-6 score improvement	0.7 (0.0–1.5)	0.3 (−0.7–0.8)	0.8 (0.0–1.7)	<0.001
EuroQoL utility	0.02 (−0.07–0.15)	0.01 (−0.09–0.12)	0.02 (−0.06–0.16)	0.306
Exacerbations (prior year) (%)	−75.0 (−100.0– −33.3)	−54.2 (−100.0–0.0)	−75.0 (−100.0– −40.0)	<0.001
ED/hospitalisation (prior year) (%)	−100.0 (−100.0– −66.7)	−100.0 (−100.0– −25.0)	−100.0 (−100.0– −66.7)	0.030
Blood eosinophil count (×10^9^ L^−1^)	−73.3 (−95.1–0.0)	0.0 (−50.0–67.9)	−80.0 (−97.7– −27.9)	<0.001
*F*_ENO_ (ppb)	−8.0 (−41.4–43.5)	−5.7 (−34.4–30.8)	−8.6 (−42.2–49.4)	0.671
FEV_1_ (mL)	20.0 (−200.0–340.0)	0.0 (−200.0–285.0)	30.0 (−200.0–350.0)	0.279
**mOCS change**				0.003
Discontinue	129 (20.2)	13 (16.0)	116 (20.8)	
Decrease dose	301 (47.2)	27 (33.3)	274 (49.2)	
Maintain dose	148 (23.2)	29 (35.8)	119 (21.4)	
Increase dose	60 (9.4)	12 (14.8)	48 (8.6)	
**Clinically important differences**				
ACQ-6 improvement	523 (62.5)	59 (41.8)	464 (66.7)	<0.001
Exacerbation reduction	746 (69.0)	117 (60.0)	629 (71.0)	0.003
Unscheduled care reduction	915 (85.5)	151 (77.0)	764 (87.4)	<0.001
FEV_1_	402 (42.5)	63 (37.5)	339 (43.6)	0.149
OCS	738 (64.9)	143 (67.1)	595 (64.4)	0.450
**Positive outcomes (n)**	3 (2–4)	3 (2–4)	3 (2–4)	<0.001
0	18 (2.7)	1 (0.9)	17 (3.0)	
1	57 (8.4)	14 (12.6)	43 (7.6)	
2	125 (18.4)	32 (28.8)	93 (16.4)	
3	172 (25.4)	31 (27.9)	141 (24.9)	
4	201 (29.6)	26 (23.4)	175 (30.9)	
5	105 (15.5)	7 (6.3)	98 (17.3)	

Data are presented as n, median (interquartile range), mean±sd or n (%), unless otherwise stated. BMI: body mass index; ED: emergency department; *F*_ENO_: exhaled nitric oxide fraction; FEV_1_: forced expiratory volume in 1 s; FVC: forced vital capacity; ACQ-6: Asthma Control Questionnaire-6; mOCS: maintenance oral corticosteroids. Definitions of clinically important differences: ACQ-6 improvement ≥0.5 or well controlled (ACQ-6 ≤0.75), exacerbation reduction ≥50% or no exacerbations, unscheduled care reduction ≥50% or no unscheduled care, FEV_1_ increase ≥100 mL and OCS dose decrease ≥50% or not receiving mOCS at follow-up.

We observed greater improvement among those receiving biologics. In particular, they experienced a larger reduction in symptoms (ACQ-6 improvement 0.8 *versus* 0.3; p<0.001) and exacerbations (75% *versus* 54% reduction; p<0.001). A larger proportion of the patients on biologics discontinued their mOCS (20.8% *versus* 16.0%; p=0.003) compared with those not receiving biologics. A much larger proportion of the patients on biologics also experienced material improvement across all five domains (17.3% *versus* 6.3%; p<0.001) compared with those not receiving biologics (tables 1 and 2, and supplementary figure E1). However, it is important to note that clinically important benefits were still observed among a substantial proportion of patients who did not receive a biologic, with over half (57.6%) showing material improvement across at least three domains.

#### Potential disparities by demographic factors

**FIGURE 1:**
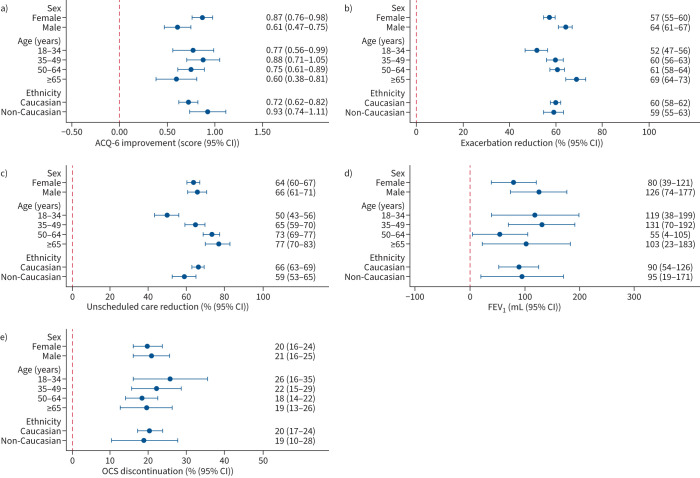
Improvements across the complete patient cohort, stratified by patient demographic variables: a) Asthma Control Questionnaire-6 (ACQ-6), b) exacerbation reduction, c) unscheduled care (emergency department attendances and hospitalisation events) reduction, d) forced expiratory volume in 1 s (FEV_1_) and e) oral corticosteroid (OCS) discontinuation. All models are adjusted for sex, age category, ethnicity, biologic therapy, year of baseline assessment, hospital site and base level of the relevant outcome of interest.

In general, considerable benefits were observed across all demographic groups, with little evidence of substantial differences after accounting for potential confounders (figure 1). At the population level, all groups had a minimum ACQ-6 decrease of 0.6, 50% reduction in exacerbations and unscheduled care, 55 mL increase in FEV_1_, and a 18% OCS discontinuance rate. However, we did observe some potentially important differences between groups. Females achieved a larger ACQ-6 improvement compared with males (0.87 *versus* 0.61; p=0.004), although this trend was reversed in exacerbation reduction (57% *versus* 64%; p=0.001). Variation was also observed by age category, with patients aged ≥65 years demonstrating larger reductions in exacerbations (69% *versus* 52%; p<0.001) and unscheduled care use (77% *versus* 50%; p<0.001) when compared with those aged 18–34 years (figure 1 and supplementary table E2). Importantly, this larger improvement among the older-aged patients was observed despite substantially reduced exacerbation and healthcare utilisation at baseline (supplementary table E3).

#### Potential disparities by centre

**FIGURE 2:**
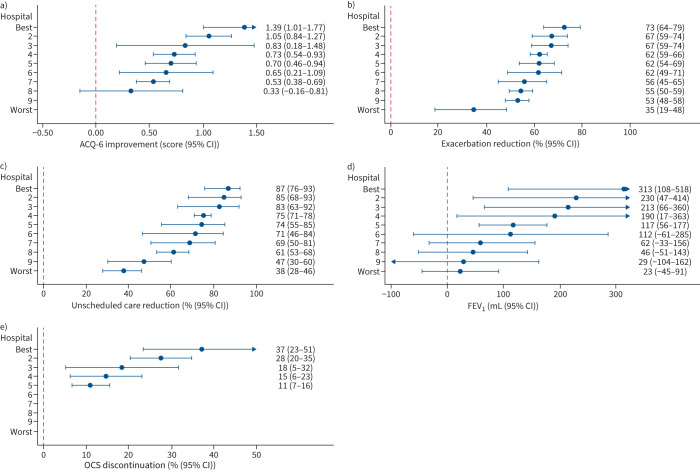
Differences in patient improvements, stratified by hospital site: a) Asthma Control Questionnaire-6 (ACQ-6), b) exacerbation reduction, c) unscheduled care (emergency department attendances and hospitalisation events) reduction, d) forced expiratory volume in 1 s (FEV_1_) and e) oral corticosteroid (OCS) discontinuation. All models are adjusted for sex, age category, ethnicity, biologic therapy, year of baseline assessment, hospital site and base level of the relevant outcome of interest. Analysis by site was restricted to hospitals with ≥30 patients per reported outcome. The terms “best” and “worst” refer to the magnitude of change for each outcome measured and are not consistent across panels.

Improvements were observed across all sites; however, the magnitude of these improvements differed substantially, even after adjustment for covariates (figure 2). For example, although seven out of eight sites achieved a mean ACQ-6 improvement >0.5, the improvements ranged from 1.39 (95% CI 1.01–1.77) to 0.33 (95% CI −0.16–0.81) between the best and worst performing hospitals (p=0.002). Similar trends were observed for reductions in exacerbations and unscheduled care use where, despite substantial improvements across all hospitals of ≥35%, 2-fold differences persisted. Substantial variation was also observed in the proportion of patients discontinuing mOCS, ranging from 11% (95% CI 7–16%) in the lowest hospital to 37% (95% CI 23–51%) in the highest (p<0.001).

### Sensitivity analyses

Omitting biologic therapy use from our models led to small changes in estimates and did not alter our conclusions (supplementary table E2). A much larger proportion (48.4%) of patients met our expanded definition of OCS discontinuance, which included those receiving ≤5 mg at follow-up; however, there was no variation by demographic groups and the magnitude of interhospital variation was similar to our primary analysis (supplementary figure E2).

## Discussion

This large study of 1140 severe asthma patients demonstrates that assessment and management by severe asthma specialists leads to substantial benefits for the majority of patients, including reduced symptoms, healthcare utilisation and mOCS. Benefits were greater among those receiving biologics; however, we also observed clinically meaningful improvement among the majority of patients who did not receive these medications. The magnitude of benefits was generally consistent across demographic groups; however, there was some evidence of a larger improvement among older-aged patients. Significant variation existed across hospital sites for all outcomes.

The BTS and ERS/ATS have highlighted the need for patients with difficult or severe asthma to be systematically evaluated by specialists [4, 5]. There are several potential benefits of this approach, including confirmation of diagnosis, identification of the mechanism driving symptoms and assessment of adherence to maintenance medications. Although there is no standardised pathway, assessment may lead to a broad range of potentially effective interventions, including changes to treatment regimen, adherence counselling, psychological therapy, physiotherapy referral or biologic therapy initiation. Our findings that specialist assessment is associated with substantially improved outcomes highlights the importance of appropriate referral from primary and secondary care, particularly as biologic therapies can only be prescribed by specialists within the UK. In that context, recent evidence demonstrating that a large number of patients with potential severe asthma are hidden within UK primary care is concerning [23].

Our findings are consistent with other evidence from the USA, France, Australia and the Netherlands [7–13] which demonstrated that specialist assessment can lead to improved asthma control, reduced exacerbations and a lowering of mOCS dose. Our results are also in broad agreement with evidence from the UK [24, 25], including within the most recent study by Gibeon *et al.* [14] that used data collected from UK severe asthma centres between 2009 and 2010. However, we reported a larger improvement in median ACQ-6 score (1.0 *versus* 0.6) and a lower proportion of patients with an exacerbation (66% *versus* 77%) or hospital admission (17% *versus* 33%) in the year prior to follow-up. While the study by Gibeon *et al.* [14] did not report any reduction in median blood eosinophil level at follow-up, we reported median reductions of 73% across the cohort, driven by 80% reductions among those receiving biologics, which suggests that these advances are likely driven by a reduction in type 2 inflammatory pathways due to the widespread use of anti-interleukin (IL)-5 and anti-IL-5 receptor biologic therapy.

Our findings that improvements were observed similarly across demographic groups are reassuring, particularly given recent findings of substantially higher asthma morbidity among ethnic minority groups presenting to UK severe asthma services [16]. There was some evidence that older patients had a larger reduction in exacerbations and unscheduled care utilisation than younger patients. The reasons for this remain unclear; however, it does not appear to be related to higher healthcare utilisation at baseline. It is known that adherence often deteriorates after biologic commencement, which can reduce the effectiveness of these medications [26]. It may be that older patients, who are known to have better adherence across all severities of asthma, are less susceptible to this phenomenon [27]. Differences in asthma phenotype have also been related to treatment response and, naturally, the early-onset phenotype is likely to be more prevalent among younger adults referred to specialist clinics [28, 29].

Our findings of geographic variation in asthma outcomes require further investigation. It has been shown elsewhere that the patients presenting to these specialist centres vary substantially in terms of demographic and clinical characteristics [30]; however, it is unlikely that this is driving all of the differences observed in our study and may instead reflect regional differences in referral practices or care pathways. In particular, wide variation in mOCS discontinuation rates may reflect differences in local protocols or variation in biologic prescribing patterns. It should be noted that our data were collected before publication of the PONENTE study, which demonstrated the effectiveness of a personalised dosage reduction algorithm among patients initiating benralizumab and might be expected to change mOCS tapering practices going forwards [22]. The UKSAR was established to provide data that would support quality improvement in severe asthma management. In response to potential variation among patient outcomes, the UK severe asthma community continues to undertake initiatives which share best practice among service providers [31].

Our study is novel, being the first to investigate potential disparities in specialist severe asthma care outcomes, and is at least three times larger than other similar analyses. It is also the first to investigate this issue within the “biologic era” of UK severe asthma treatment and uses real-world data from the majority of specialist severe asthma centres in the UK. The primary weakness of our study is the lack of a comparison group, meaning that some of the improvements observed in our study could be due to regression to the mean [32]. However, due to the magnitude and consistency of the effects observed we do not think this statistical phenomenon is the primary driver of our results. A randomised controlled trial is required to fully address this issue; however, given the consensus that specialist assessment of severe asthma is beneficial, this is unlikely to be ethically viable. It is unclear if our results can be generalised to countries outside the UK due to significant heterogeneity between severe asthma populations and treatment patterns worldwide [33, 34].

In conclusion, specialist assessment and management leads to substantially improved patient outcomes, which are broadly consistent across demographic groups and are not restricted to those receiving biologic therapy. The magnitude of these improvements is larger than those observed in previous studies of UK severe asthmatic subjects, which is likely mediated by a reduction in type 2 inflammatory pathways due to the widespread use of anti-IL-5 and anti-IL-5 receptor biologic therapy. Significant variation was observed between hospitals, which requires further investigation.

## Supplementary material

10.1183/13993003.00660-2022.Supp1 **Please note:** supplementary material is not edited by the Editorial Office, and is uploaded as it has been supplied by the author.
Supplementary material ERJ-00660-2022.Supplement

## Shareable PDF

10.1183/13993003.00660-2022.Shareable1 This one-page PDF can be shared freely online.
Shareable PDF ERJ-00660-2022.Shareable

## References

[1] Vos T, Flaxman AD, Naghavi M, et al. Years lived with disability (YLDs) for 1160 sequelae of 289 diseases and injuries 1990–2010: a systematic analysis for the Global Burden of Disease Study 2010. Lancet 2012; 380: 2163–2196. doi:10.1016/S0140-6736(12)61729-2 23245607 PMC6350784

[2] Global Initiative for Asthma (GINA). Global Strategy for Asthma Management and Prevention. 2021. Available from: http://ginasthma.org/

[3] Global Initiative for Asthma (GINA). Difficult-to-treat & severe asthma in adolescent and adult patients. Diagnosis and management. 2019. Available from: http://ginasthma.org/

[4] Scottish Intercollegiate Guidelines Network/British Thoracic Society. SIGN 158 British Guideline on the Management of Asthma: A National Clinical Guideline. 2019. www.sign.ac.uk/our-guidelines/british-guideline-on-the-management-of-asthma Date last accessed: 2 July 2022.

[5] Chung KF, Wenzel SE, Brozek JL, et al. International ERS/ATS guidelines on definition, evaluation and treatment of severe asthma. Eur Respir J 2014; 43: 343–373. doi:10.1183/09031936.00202013 24337046

[6] Barnes PJ, Chung KF. Difficult asthma. Br Med J 1989; 299: 695–698. doi:10.1136/bmj.299.6701.695 2508876 PMC1837483

[7] van der Meer AN, Pasma H, Kempenaar-Okkema W, et al. A 1-day visit in a severe asthma centre: effect on asthma control, quality of life and healthcare use. Eur Respir J 2016; 48: 726–733. doi:10.1183/13993003.00220-2016 27338198

[8] Irwin RS, Curley FJ, French CL. Difficult-to-control asthma. Contributing factors and outcome of a systematic management protocol. Chest 1993; 103: 1662–1669. doi:10.1378/chest.103.6.1662 8404082

[9] Bratton DL, Price M, Gavin L, et al. Impact of a multidisciplinary day program on disease and healthcare costs in children and adolescents with severe asthma: a two-year follow-up study. Pediatr Pulmonol 2001; 31: 177–189. doi:10.1002/ppul.1027 11276130

[10] Krupp NL, Weist A, Fiscus CD, et al. Efficacy, cost effectiveness, and sustainability of a pediatric high risk asthma clinic. Pediatr Pulmonol 2018; 53: 538–543. doi:10.1002/ppul.23967 29484838

[11] Tay TR, Lee J, Radhakrishna N, et al. A structured approach to specialist-referred difficult asthma patients improves control of comorbidities and enhances asthma outcomes. J Allergy Clin Immunol Pract 2017; 5: 956–964. doi:10.1016/j.jaip.2016.12.030 28284780

[12] Begne C, Justet A, Dupin C, et al. Evaluation in a severe asthma expert center improves asthma outcomes regardless of step-up in asthma therapy. J Allergy Clin Immunol Pract 2020; 8: 1439–1442. doi:10.1016/j.jaip.2019.10.026 31706047

[13] Denton E, Lee J, Tay T, et al. Systematic assessment for difficult and severe asthma improves outcomes and halves oral corticosteroid burden independent of monoclonal biologic use. J Allergy Clin Immunol Pract 2020; 8: 1616–1624. doi:10.1016/j.jaip.2019.12.037 31954193

[14] Gibeon D, Heaney LG, Brightling CE, et al. Dedicated severe asthma services improve health-care use and quality of life. Chest 2015; 148: 870–876. doi:10.1378/chest.14-3056 25789861

[15] Busby J, Price D, Al-Lehebi R, et al. Impact of socioeconomic status on adult patients with asthma: a population-based cohort study from UK primary care. J Asthma Allergy 2021; 14: 1375–1388. doi:10.2147/JAA.S326213 34785911 PMC8591110

[16] Busby J, Heaney LG, Brown T, et al. Ethnic differences in severe asthma clinical care and outcomes: an analysis of United Kingdom primary and specialist care. J Allergy Clin Immunol Pract 2022; 10: 495–505. doi:10.1016/j.jaip.2021.09.034 34626858

[17] Redmond C, Akinoso-Imran AQ, Heaney LG, et al. Socioeconomic disparities in asthma health care utilization, exacerbations, and mortality: a systematic review and meta-analysis. J Allergy Clin Immunol 2022; 149: 1617–1627. doi:10.1016/j.jaci.2021.10.007 34673047

[18] Chowdhury NU, Guntur VP, Newcomb DC, et al. Sex and gender in asthma. Eur Respir Rev 2021; 30: 210067. doi:10.1183/16000617.0067-2021 34789462 PMC8783601

[19] Jackson DJ, Busby J, Pfeffer PE, et al. Characterisation of patients with severe asthma in the UK Severe Asthma Registry in the biologic era. Thorax 2021; 76: 220–227. doi:10.1136/thoraxjnl-2020-215168 33298582 PMC7892381

[20] Heaney LG, Brightling CE, Menzies-Gow A, et al. Refractory asthma in the UK: cross-sectional findings from a UK multicentre registry. Thorax 2010; 65: 787–794. doi:10.1136/thx.2010.137414 20805172 PMC2975949

[21] Williams R. Using the margins command to estimate and interpret adjusted predictions and marginal effects. Stata J 2012; 12: 308–331. doi:10.1177/1536867X1201200209

[22] Menzies-Gow A, Gurnell M, Heaney LG, et al. Oral corticosteroid elimination via a personalised reduction algorithm in adults with severe, eosinophilic asthma treated with benralizumab (PONENTE): a multicentre, open-label, single-arm study. Lancet Respir Med 2022; 10: 47–58. doi:10.1016/S2213-2600(21)00352-0 34619104

[23] Ryan D, Heatley H, Heaney LG, et al. Potential severe asthma hidden in UK primary care. J Allergy Clin Immunol Pract 2021; 9: 1612–1623. doi:10.1016/j.jaip.2020.11.053 33309935

[24] Patil VK, Townshend C, Mitchell F, et al. An outreaching model of tertiary difficult asthma care reduces adverse asthma outcomes and healthcare utilisation costs. Eur Respir J 2016; 47: 1857–1860. doi:10.1183/13993003.01689-2015 27009175

[25] Sweeney J, Brightling CE, Menzies-Gow A, et al. Clinical management and outcome of refractory asthma in the UK from the British Thoracic Society Difficult Asthma Registry. Thorax 2012; 67: 754–756. doi:10.1136/thoraxjnl-2012-201869 22581823 PMC3402747

[26] Ancona G, Kavanagh J, Roxas C, et al. Adherence to corticosteroids and clinical outcomes in mepolizumab therapy for severe asthma. Eur Respir J 2020; 55: 1902259. doi:10.1183/13993003.02259-2019 32060061

[27] Dima AL, Hernandez G, Cunillera O, et al. Asthma inhaler adherence determinants in adults: systematic review of observational data. Eur Respir J 2015; 45: 994–1018. doi:10.1183/09031936.00172114 25504997

[28] Haldar P, Pavord ID, Shaw DE, et al. Cluster analysis and clinical asthma phenotypes. Am J Respir Crit Care Med 2008; 178: 218–224. doi:10.1164/rccm.200711-1754OC 18480428 PMC3992366

[29] de Nijs SB, Venekamp LN, Bel EH. Adult-onset asthma: is it really different? Eur Respir Rev 2013; 22: 44–52. doi:10.1183/09059180.00007112 23457164 PMC9487439

[30] Busby J, Matthews JG, Chaudhuri R, et al. Factors affecting adherence with treatment advice in a clinical trial of patients with severe asthma. Eur Respir J 2022; 59: 2100768. doi:10.1183/13993003.00768-2021 34561291 PMC9202483

[31] Oxford Academic Health Science Network. AAC Consensus Pathway for Uncontrolled and Severe Asthma 2022. 2022. www.oxfordahsn.org/our-work/asthma-biologics-toolkit/aac-consensus-pathway-for-management-of-uncontrolled-asthma-in-adults Date last accessed: 2 July 2022.

[32] Barnett AG, van der Pols JC, Dobson AJ. Regression to the mean: what it is and how to deal with it. Int J Epidemiol 2005; 34: 215–220. doi:10.1093/ije/dyh299 15333621

[33] Porsbjerg CM, Menzies-Gow AN, Tran TN, et al. Global variability in administrative approval prescription criteria for biologic therapy in severe asthma. J Allergy Clin Immunol Pract 2022; 10: 1202–1216. doi:10.1016/j.jaip.2021.12.027 34990866

[34] Wang E, Wechsler ME, Tran TN, et al. Characterization of severe asthma worldwide: data from the International Severe Asthma Registry. Chest 2020; 157: 790–804. doi:10.1016/j.chest.2019.10.053 31785254

