# Supplementary material for: Benefits of specialist severe asthma management: demographic and geographic disparities
Source: Eur Respir J. 2022 Dec 15;60(6):2200660. doi: 10.1183/13993003.00660-2022 (PMC9753476; doi:10.1183/13993003.00660-2022)
Supplement: Supplementary file 1 [file ERJ-00660-2022.Supplement.pdf]

Supplement

Table E1 - Data completeness

Characteristic	Baseline	Follow-up
**Age at assessment**	1,140 (100%)	N/A
**Gender**	1,140 (100%)	N/A
**Ethnicity**	1,136 (99.6%)	N/A
**BMI**	1,135 (99.6%)	903 (79.2%)
**Smoking status**	1,116 (97.9%)	N/A
**FEV_1**	1,124 (98.6%)	955 (83.8%)
**FVC**	1,113 (97.6%)	911 (79.9%)
**FEV_1/FVC**	1,113 (97.6%)	910 (79.8%)
**ACQ6**	987 (86.6%)	929 (81.5%)
**Exacerbations (Prior year)**	1,120 (98.3%)	1,101 (96.6%)
**ED Attendance (Prior year)**	1,120 (98.3%)	1,089 (95.5%)
**Hospital Admissions (Prior year)**	1,127 (98.9%)	1,090 (95.6%)
**Blood Eosinophil Count**	1,119 (98.2%)	705 (61.8%)
**FeNO**	898 (78.8%)	583 (51.1%)
**IGE**	1,100 (96.5%)	N/A
**mOCS**	1,134 (99.5%)	1,138 (99.8%)
**mOCS dose**	637 (99.7%)	585 (99.7%)
**ICS dose**	1,062 (93.2%)	N/A
**Biologic Therapy**	NA	1,140 (100%)

Figure E1 – Bar chart depicting the representative proportion of patients that achieved improvement across five patient outcomes*

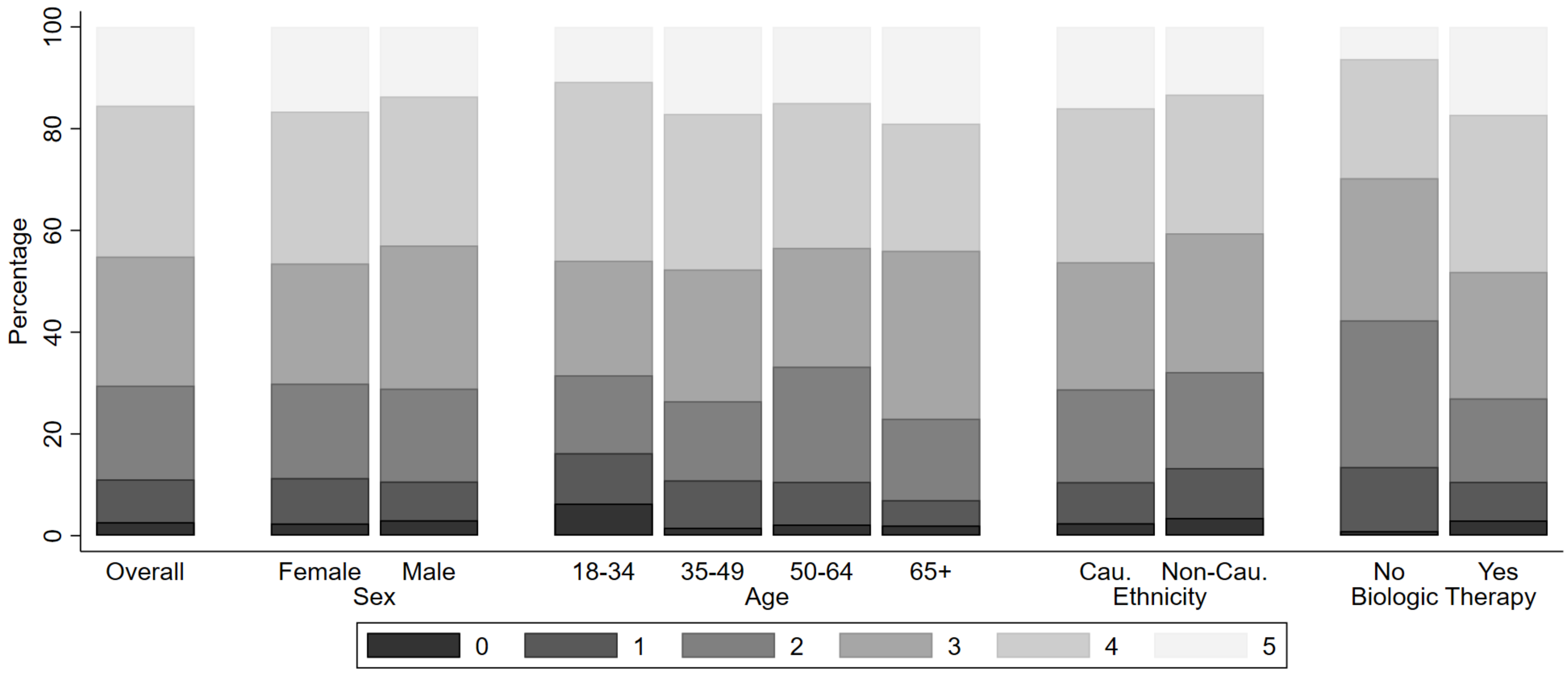

***Outcomes included:** ACQ improvement of 0.5 or greater or Well Controlled (ACQ <=0.75), an exacerbation reduction >=50% or No Exacerbations, a 50% or greater reduction in unscheduled care (ED attendance/hospitalisation) or No Unscheduled Secondary Care, an FEV_1 increase of 100ml or greater, an OCS dose reduction of 50% or greater (or not receiving OCS at follow-up appointment).

Abbreviation: Cau (Caucasian)

Table E2 – Comparison of specialist referral benefit by demographic factors and biologic treatment status

Exposure	ACQ6 Score [a]				Exacerbations [b]			
	**Univariate**	**Multivariate (No biologic)**	**Multivariate**	**P-Value**	**Univariate**	**Multivariate (No biologic)**	**Multivariate**	**P-Value**
**Sex**	**N=1079**	**N=1075**	**N=1075**		**N=1140**	**N=1136**	**N=1136**	
Female	Ref	Ref	Ref	.	Ref	Ref	Ref	.
Male	0.24 (0.06,0.42)	0.24 (0.05,0.42)	0.26 (0.08,0.44)	0.004	0.83 (0.75,0.91)	0.83 (0.75,0.91)	0.84 (0.76,0.93)	0.001
**Age At First Assessment (Years)**	**N=1079**	**N=1075**	**N=1075**		**N=1140**	**N=1136**	**N=1136**	
18-34	Ref	Ref	Ref	.	Ref	Ref	Ref	.
35-49	-0.13 (-0.41,0.15)	-0.13 (-0.41,0.15)	-0.10 (-0.38,0.17)	0.457	0.82 (0.72,0.94)	0.82 (0.72,0.94)	0.83 (0.73,0.95)	0.008
50-64	-0.01 (-0.27,0.25)	-0.01 (-0.27,0.25)	0.02 (-0.23,0.28)	0.850	0.80 (0.71,0.91)	0.80 (0.71,0.91)	0.82 (0.72,0.92)	0.001
65+	0.14 (-0.17,0.45)	0.14 (-0.17,0.45)	0.18 (-0.13,0.48)	0.258	0.63 (0.54,0.75)	0.63 (0.54,0.75)	0.65 (0.55,0.77)	0.000
**Ethnicity**	**N=1075**	**N=1075**	**N=1075**		**N=1136**	**N=1136**	**N=1136**	
Caucasian	Ref	Ref	Ref	.	Ref	Ref	Ref	.
Non-Caucasian	-0.12 (-0.33,0.10)	-0.12 (-0.33,0.10)	-0.20 (-0.41,0.01)	0.063	1.05 (0.93,1.18)	1.05 (0.93,1.18)	1.02 (0.91,1.15)	0.732

Exposure	Unscheduled Care Use [b]				OCS Discontinuation [c]			
	**Univariate**	**Multivariate (No biologic)**	**Multivariate**	**P-Value**	**Univariate**	**Multivariate (No biologic)**	**Multivariate**	**P-Value**
**Sex**	**N=1139**	**N=1135**	**N=1135**		**N=637**	**N=635**	**N=635**	
Female	Ref	Ref	Ref	.	Ref	Ref	Ref	.
Male	0.94 (0.79,1.12)	0.94 (0.79,1.12)	0.94 (0.79,1.12)	0.489	1.04 (0.69,1.56)	1.07 (0.71,1.62)	1.07 (0.71,1.62)	0.751
**Age At First Assessment (Years)**	**N=1139**	**N=1135**	**N=1135**		**N=637**	**N=635**	**N=635**	
18-34	Ref	Ref	Ref	.	Ref	Ref	Ref	.
35-49	0.70 (0.57,0.85)	0.70 (0.57,0.85)	0.70 (0.58,0.86)	0.001	0.83 (0.41,1.66)	0.81 (0.40,1.63)	0.80 (0.39,1.62)	0.534
50-64	0.53 (0.44,0.65)	0.54 (0.44,0.65)	0.53 (0.44,0.65)	0.000	0.62 (0.33,1.17)	0.62 (0.33,1.19)	0.62 (0.32,1.18)	0.143
65+	0.45 (0.33,0.61)	0.45 (0.33,0.61)	0.46 (0.34,0.62)	0.000	0.68 (0.33,1.43)	0.68 (0.32,1.42)	0.67 (0.32,1.42)	0.297
**Ethnicity**	**N=1135**	**N=1135**	**N=1135**		**N=635**	**N=635**	**N=635**	
Caucasian	Ref	Ref	Ref	.	Ref	Ref	Ref	.
Non-Caucasian	1.23 (1.03,1.46)	1.23 (1.03,1.46)	1.21 (1.01,1.45)	0.034	0.87 (0.45,1.69)	0.89 (0.45,1.73)	0.90 (0.46,1.77)	0.761

Table E2 Footnote: Univariate analysis adjusts for hospital site, year of baseline assessment and time. Multivariate analysis (no biologic) adjusts for hospital site, year of baseline assessment, time, sex, age and ethnicity. Multivariate analysis adjusts for hospital site, year of baseline assessment, time, sex, age, ethnicity, and biologic therapy. All models included an interaction term between time and the variable of interest.

[a] Mean difference; [b] Incidence rate ratio; [c] Odds ratio

Figure E2 – OCS discontinuation, defined as complete withdrawal of OCS or remaining on 5mg or less at annual review, stratified by patient demographic variables and hospital site

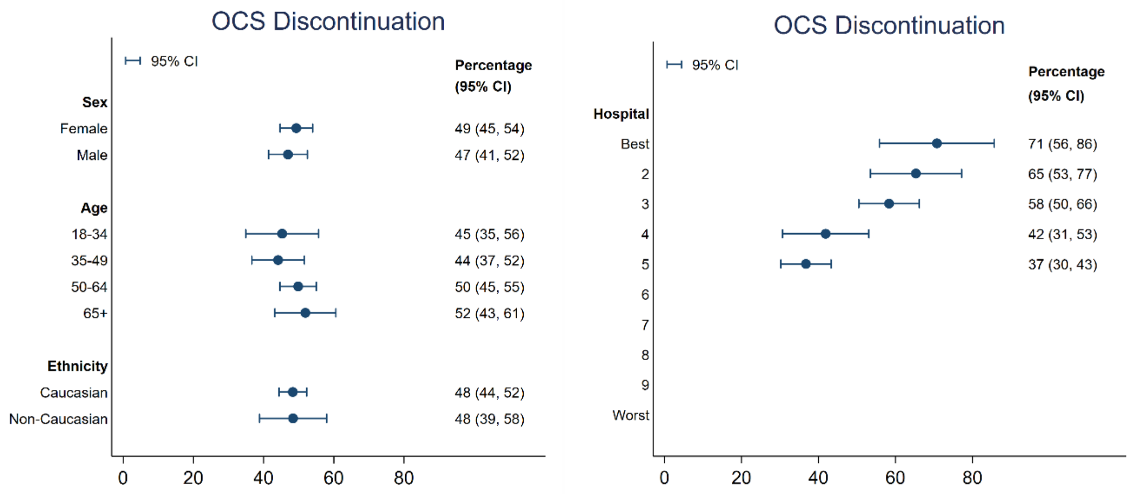

*All models are adjusted for sex, age category, ethnicity, biologic therapy, year of baseline assessment, hospital site, and base level of the relevant outcome of interest

**Analysis by site was restricted to hospitals with 30 or more patients per reported outcome.

***The terms best and worst refer to the magnitude of change for each outcome measured and are not consistent across panels.

Abbreviation: OCS (Oral corticosteroids)

Table E3 – Baseline patient characteristics stratified by age group at first assessment

	18-34	35-49	50-64	65+	P-value
**Number of Patients**	189	300	461	190	
**Age At First Assessment (Years)**	26.9 (4.6)	43.4 (4.2)	56.5 (4.2)	70.9 (6.9)	<0.001
**Age of Onset (Years)**	10.5 (8.3)	19.4 (13.6)	29.2 (18.5)	40.5 (22.5)	<0.001
**Gender**					<0.001
Female	142 (75.1%)	187 (62.3%)	270 (58.6%)	97 (51.1%)	
Male	47 (24.9%)	113 (37.7%)	191 (41.4%)	93 (48.9%)	
**Ethnicity**					0.007
Caucasian	152 (80.4%)	225 (75.3%)	366 (79.9%)	170 (89.5%)	
Non-Caucasian	37 (19.6%)	74 (24.7%)	92 (20.1%)	20 (10.5%)	
**BMI (kg/m2)**	29.9 (7.9)	31.1 (7.5)	31.2 (6.9)	29.6 (5.4)	0.013
**Smoking Status**					<0.001
Never smoked	137 (74.5%)	197 (67.0%)	293 (64.8%)	129 (69.4%)	
Ex-smoker	31 (16.8%)	85 (28.9%)	150 (33.2%)	55 (29.6%)	
Current smoker	16 (8.7%)	12 (4.1%)	9 (2.0%)	2 (1.1%)	
**Atopic Disease**	143 (75.7%)	187 (63.2%)	224 (49.0%)	74 (40.0%)	<0.001
**FEV1 (ml)**	2416.6 (801.6)	2159.6 (805.7)	1845.1 (711.5)	1709.3 (621.6)	<0.001
**FEV1 (% Predicted)**	70.3 (20.5)	67.1 (21.2)	64.3 (21.2)	67.7 (20.1)	0.008
**FVC (ml)**	3544.8 (934.2)	3348.7 (1061.6)	2979.5 (975.4)	2866.3 (954.6)	<0.001
**FVC (% Predicted)**	87.7 (16.5)	84.0 (19.1)	82.1 (18.9)	87.1 (19.9)	0.001
**FEV1/FVC**	67.6 (13.4)	64.7 (13.7)	62.7 (23.6)	60.7 (13.9)	0.002
**ACQ6 Score**	3.3 (2.3,4.3)	3.2 (2.2,4.2)	3.0 (1.8,3.8)	2.3 (1.3,3.2)	<0.001
**Uncontrolled Asthma (ACQ6>1.5)**	155 (90.6%)	214 (84.3%)	330 (82.9%)	112 (68.3%)	<0.001
**EuroQoL Utility**	0.73 (0.46,0.89)	0.74 (0.53,0.89)	0.70 (0.43,0.85)	0.78 (0.51,0.89)	0.483
**Exacerbations (Prior Year)**	6 (4,10)	5 (3,7)	4 (3,8)	5 (2,6)	<0.001
**Any ED Attendance (Prior Year)**	104 (55.9%)	129 (43.6%)	148 (33.0%)	54 (28.7%)	<0.001
**Any Hospital Admissions (Prior Year)**	96 (51.3%)	115 (38.9%)	156 (34.3%)	70 (37.0%)	0.008
**Invasive Ventilations (Ever)**	31 (16.8%)	31 (10.5%)	47 (10.4%)	8 (4.3%)	0.016
**Eczema**	11 (5.8%)	7 (2.3%)	6 (1.3%)	3 (1.6%)	0.006
**Nasal Polyps**	24 (12.7%)	54 (18.0%)	98 (21.3%)	35 (18.4%)	0.086
**Gastro-oesophageal Reflux**	29 (15.3%)	50 (16.7%)	87 (18.9%)	41 (21.6%)	0.375
**Depression or Anxiety**	23 (12.2%)	33 (11.0%)	48 (10.4%)	5 (2.6%)	0.004
**Blood Eosinophil Count (N/109L)**	0.40 (0.20,0.60)	0.41 (0.20,0.70)	0.33 (0.20,0.60)	0.31 (0.15,0.55)	0.009
**Highest Blood Eosinophil Count (N/109L)**	0.70 (0.40,1.10)	0.70 (0.40,1.18)	0.66 (0.40,1.00)	0.68 (0.44,1.10)	0.578
**FeNO (ppb)**	49 (21,87)	39 (20,75)	41 (25,69)	38 (19,63)	0.276
**IGE (IU/mL)**	204 (85,551)	152 (61,432)	147 (47,389)	145 (44,466)	0.013

**mOCS**	79 (41.8%)	156 (52.3%)	294 (64.2%)	110 (58.2%)	<0.001
**mOCS (prednisolone equivalent/mg)**	10 (10,20)	10 (10,20)	10 (8,15)	10 (5,10)	<0.001
**ICS Dose (BDP equivalent/ug)**	2000 (1600,2000)	2000 (1600,2000)	2000 (1600,2000)	2000 (1600,2000)	0.592
**LAMA**	111 (58.7%)	162 (54.4%)	254 (56.3%)	119 (63.3%)	0.098
**Theophylline**	45 (23.9%)	69 (23.2%)	129 (28.4%)	36 (18.9%)	0.075
**Leukotriene Receptor Antagonists**	107 (59.4%)	148 (50.7%)	224 (49.7%)	89 (47.6%)	0.095
**Maintenance Macrolides**	13 (7.2%)	12 (4.1%)	43 (9.4%)	14 (7.5%)	0.029
**Nebuliser**	42 (22.6%)	51 (17.0%)	102 (22.8%)	37 (19.8%)	0.029
**Biologic Therapy**	142 (75.1%)	242 (80.7%)	382 (82.9%)	161 (84.7%)	0.071
**Biological Therapy Name**					0.048
Mepolizumab	71 (52.6%)	151 (64.0%)	260 (69.9%)	109 (69.4%)	
Benralizumab	32 (23.7%)	49 (20.8%)	63 (16.9%)	33 (21.0%)	
Omalizumab	31 (23.0%)	36 (15.3%)	48 (12.9%)	15 (9.6%)	
Dupilumab	1 (0.7%)	0 (0.0%)	1 (0.3%)	0 (0.0%)	

Footnote: Abbreviations: FEV1 (Forced expiratory volume in the first second), FVC (Forced vital capacity), ACQ6 (Asthma Control Questionnaire 6), ED (Emergency Department), FeNO (Fractional exhaled nitric oxide), IGE (Immunoglobulin E), mOCS (Maintenace oral corticosteroids), ICS (Inhaled corticosteroids), LAMA (Long-acting muscarinic antagonists), SABA (Short-acting beta-agonists)
