# Supplementary material for: Benefits of specialist severe asthma management: demographic and geographic disparities
Source: Eur Respir J. 2022 Dec 15;60(6):2200660. doi: 10.1183/13993003.00660-2022 (PMC9753476; doi:10.1183/13993003.00660-2022)
Supplement: Supplementary file 2 [file ERJ-00660-2022.Shareable.pdf]

EUROPEAN RESPIRATORY JOURNAL
ORIGINAL RESEARCH ARTICLE
C. REDMOND ET AL.

SHAREABLE PDF

Benefits of specialist severe asthma management: demographic and geographic disparities

Charlene Redmond [1], Liam G. Heaney[1,2], Rekha Chaudhuri[3], David J. Jackson[4,5], Andrew Menzies-Gow[6], Paul Pfeffer[7] and John Busby[1] on behalf of the UK Severe Asthma Registry

[1]School of Medicine, Dentistry and Biomedical Sciences, Queen's University, Belfast, UK. [2]Belfast Health and Social Care NHS Trust, Belfast, UK. [3]Gartnavel General Hospital, Glasgow, UK. [4]Guy's Severe Asthma Centre, Guy's and St Thomas' Hospitals, London, UK. [5]School of Immunology and Microbial Sciences, King's College London, London, UK. [6]Royal Brompton and Harefield Hospitals, London, UK. [7]Barts Health NHS Trust, London, UK.

Corresponding author: John Busby (john.busby@qub.ac.uk)

Check for updates

Shareable abstract (@ERSpublications)
Specialist assessment and management of patients with severe asthma leads to substantially improved patient outcomes, which are broadly consistent across demographic groups although vary substantially across hospitals https://bit.ly/3ORDfei

Cite this article as: Redmond C, Heaney LG, Chaudhuri R, *et al*. Benefits of specialist severe asthma management: demographic and geographic disparities. *Eur Respir J* 2022; 60: 2200660 [DOI: 10.1183/13993003.00660-2022].

This single-page version can be shared freely online.

Copyright ©The authors 2022.

This version is distributed under the terms of the Creative Commons Attribution Licence 4.0.

Received: 28 March 2022
Accepted: 23 June 2022

Abstract

Background The benefits of specialist assessment and management have yet to be evaluated within the biologic era of UK severe asthma treatment, and potential disparities have not been considered.

Methods In an uncontrolled before-and-after study, we compared asthma symptoms (Asthma Control Questionnaire-6 (ACQ-6)), exacerbations, unscheduled secondary care use, lung function (forced expiratory volume in 1 s (FEV_1)) and oral corticosteroid (OCS) dose after 1 year. We compared outcomes by sex, age (18–34, 35–49, 50–64 and ⩾65 years), ethnicity (Caucasian *versus* non-Caucasian) and hospital site after adjusting for demographics and variation in biologic therapy use.

Results 1140 patients were followed-up for 1370 person-years from 12 specialist centres. At annual review, ACQ-6 score was reduced by a median (interquartile range (IQR)) of 0.7 (0.0–1.5), exacerbations by 75% (33–100%) and unscheduled secondary care by 100% (67–100%). FEV_1 increased by a median (IQR) of 20 (−200–340) mL, while OCS dose decreased for 67% of patients. Clinically meaningful improvements occurred across almost all patients, including those not receiving biologic therapy. There was little evidence of differences across demographic groups, although those aged ⩾65 years demonstrated larger reductions in exacerbations (69% *versus* 52%; $p<0.001$) and unscheduled care use (77% *versus* 50%; $p<0.001$) compared with patients aged 18–34 years. There were >2-fold differences between the best and worst performing centres across all study outcomes.

Conclusions Specialist assessment and management is associated with substantially improved patient outcomes, which are broadly consistent across demographic groups and are not restricted to those receiving biologic therapy. Significant variation exists between hospitals, which requires further investigation.
